# A compact-rigid multi-analyser for energy and angle filtering of high-resolution X-ray experiments. Part 2. Efficiency of a single-crystal-comb

**DOI:** 10.1107/S1600577522011250

**Published:** 2023-01-01

**Authors:** J.-L. Hodeau, A. Prat, N. Boudet, N. Blanc, S. Arnaud, J.-L. Hazemann, E. Lahéra, O. Proux, M. Jacquet, P.-O. Autran, C. Dejoie, P. Martinetto

**Affiliations:** a Institut Néel CNRS-UGA, 25 Avenue des Martyrs, 38042 Grenoble, France; b OSUG-FAME, CNRS-UGA-IRD-INRAe-MétéoFrance, 71 Avenue des Martyrs, 38000 Grenoble, France; c LAL, Univ. Paris-Sud XI, CNRS-IN2P3, Orsay, France; d European Synchrotron Radiation Facility, 71 Avenue des Martyrs, F-38000 Grenoble, France; University of Malaga, Spain

**Keywords:** high-resolution powder diffraction, multi-analyser-diffraction filtering, heterogeneous materials, compact-rigid multi-analyser

## Abstract

Diffraction instruments using filtering by one or several analyser crystals exist since the 1980s and 1990s at synchrotron radiation sources, but, due to its low efficiency, this filtering is little used on laboratory sources. In order to overcome this limitation, the efficiency of a small diffraction filtering multi-analyzer block (MAD block) realized with a ‘single-crystal-comb’ curved on a rigid support is demonstrated here. The geometry of this curved surface is logarithmic spiral and is optimized to allow multi-filtering over a relatively important diffraction angular range and to be also applicable over an X-ray spectral range. The efficiency of such a small rigid-compact MAD block consisting of this single-crystal-comb generating 20–50 Si(111) single-crystal blades, associated with a block of Soller collimators, is demonstrated. The angle between each crystal is 0.1°, so the measurement range of the comb is 2–5°. The geometry of this system has been optimized for operation with a synchrotron X-ray source over an energy range of 22 keV to 46 keV and could be used with laboratory X-ray sources (Ag *K*α_1_, 22.1 keV). This MAD block complements and exploits the qualities of the ‘photon-counting’ detectors which have very low intrinsic noise. Their joint efficacy is supported by powder pattern measurements of a LaB_6_ reference sample and of several heterogeneous samples of cultural heritage materials, carried out at 22 keV on the D2AM beamline at the ESRF. Their signal-to-noise ratio is excellent (1000/1) and allows the detection thresholds of the measurements (from 3–1% to 0.1%) to detect minor phases in the studies of ‘real’ heterogeneous materials to be drastically improved.

## Abstract 2

The efficiency of a small multi-analyser block of diffraction filtering, formed by a ‘single-crystal-comb’ curved on a logarithmic spiral rigid support that allows 20–50 filtering measurements in parallel, is discussed and tested on a reference powder and on complex samples. It gives an excellent signal-to-noise ratio (1000/1) and drastically improves the detection thresholds of measurements (from 3–1% to 0.1%) to detect minor phases in studies of ‘real’ heterogeneous materials.

## 1. Introduction

A difficult problem to solve for structural experiments is the identification and quantification of phases in heterogeneous samples where amorphous and crystalline phases coexist and where the presence of minor phases is important. This is the case when the samples are materials for industrial application where the properties are not related to the support material but to the contribution of its few external materials/inclusions, and also when they are cultural heritage materials where the minor phases may be the signature of the origin of the raw materials or of an ancient know-how. This underlines the need to develop easily accessible high-angular-resolution experiments to perform accurate structural analyses of complex and heterogeneous materials in order to quantify their weak signals. Such studies often use the complementarity of diffraction/scattering and spectroscopic measurements; however, one difficulty of these two types of measurements is in obtaining a good energy and directional filtering of their data in order to select only the beams emitted or scattered by a dedicated part of the object. The performance of these analyses is only fully achieved if all components of the measurement chain (beam–optics–instrument–detection) have very good performances, both in terms of angular and energy resolution and detection threshold. For this purpose, the use of a crystal analyser as ‘diffraction filter’ between the analysed object and the detector has long proved to be very effective both in spectroscopy (Zhong *et al.*, 1999 ▸; Hazemann *et al.*, 2009 ▸; Proux *et al.*, 2017 ▸) and in diffraction (Cox *et al.*, 1983 ▸; Cox, 1992 ▸; Hastings *et al.*, 1984 ▸). These analyser crystals only detect ‘good’ photons by acting as an energy bandpath filter and a ‘small-aperture slits/collimator’ filter. However, the use of such a diffraction filter for accurate measurements leads to a decrease in the intensity of the signal received by the detector and therefore to long experimental times and generally to the impossibility of using laboratory X-ray sources. Consequently, these filtering optical geometries are mainly used for experiments and structural analyses using synchrotron radiation (SR) sources, whose specificities (high flux, white beam, energy-tunable monochromatic beam, low divergence, energy resolution) make them powerful and efficient tools.

If such dedicated diffraction filters are developed to be combined with 1D or 2D detectors, several sets of data can be collected in parallel generating significant time-saving in data collection. Additionally, this combination of a ‘MAD filter’ with a 1D or 2D ‘photon-counting’ detector is very efficient to obtain a very good signal-to-noise ratio since these photon-counting detectors are sensitive to the individual measurement of each photon and provide measurements with almost zero residual noise over a fairly large area (Delpierre *et al.*, 2001 ▸; Palancher *et al.*, 2005 ▸; Basolo *et al.*, 2007 ▸; Medjoubi *et al.*, 2010 ▸; Bergamaschi *et al.*, 2010 ▸; Dyadkin *et al.*, 2016 ▸; Dejoie *et al.*, 2018 ▸; Fitch & Dejoie, 2021 ▸). These two advances allow experiments to improve their detection thresholds of weak signals and may help to overcome some of the intensity losses of diffraction filtering. Most photon-counting detectors are multi-detectors (1D or 2D), whose small pixel size (100–200 µm) combined with a fairly large distance from the sample (200–900 mm) can complement the filtering device and allows fast and accurate angular measurement of several beams emitted by the sample over a large area. Therefore, in order to overcome the limitations of the low efficiency of diffraction filtering, it is important that these 1D–2D detectors can be combined with optics using multiple analyser crystals which allow diffraction filtering in parallel (MAD optics). This underlines the interest in developing MAD assemblies containing multiple analyser crystals for powder diffraction experiments but also for fluorescence spectroscopy and inelastic scattering experiments, as has been done on several synchrotron beamlines (Toraya *et al.*, 1996 ▸; Hodeau *et al.*, 1996 ▸, 1998 ▸; Siddons *et al.*, 1998 ▸; Zhong *et al.*, 1999 ▸; Fitch, 2004 ▸; Gozzo *et al.*, 2004 ▸; Patterson *et al.*, 2005 ▸; Gozzo *et al.*, 2006 ▸; Elkaïm *et al.*, 2007 ▸; Lee *et al.*, 2008 ▸; Wang *et al.*, 2008 ▸; Tartoni *et al.*, 2008 ▸; Thompson *et al.*, 2009 ▸; Hazemann *et al.*, 2009 ▸; Peral *et al.*, 2011 ▸; Llorens *et al.*, 2012 ▸, 2014 ▸; Sitaud *et al.*, 2012 ▸; Fauth *et al.*, 2013 ▸; Ketenoglu *et al.*, 2015 ▸; Dejoie *et al.*, 2018 ▸; Schökel *et al.*, 2021 ▸; Fitch & Dejoie, 2021 ▸), and also for some laboratory experiments (Toraya, 2009 ▸; Bès *et al.*, 2018 ▸; Honkanen *et al.*, 2019 ▸).

**Figure 1:**
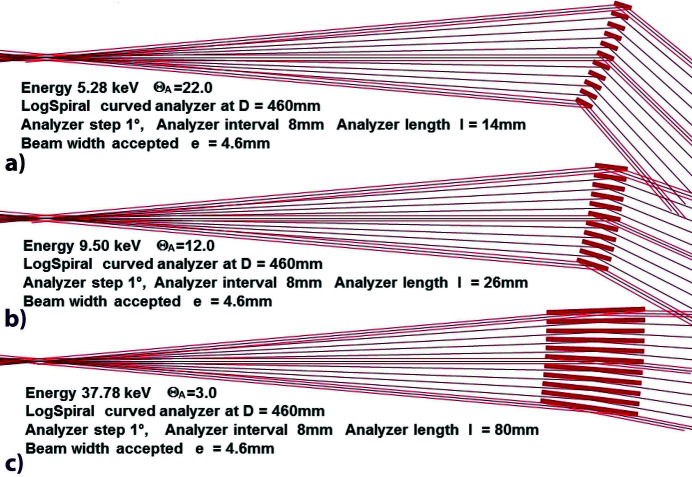
The geometric parameters of the LogSpiral curved MAD system are determined by the energy range used; the corresponding values of angles Θ_A_ and 2Θ_A_ for a Si(111) analyser and the detector determine the length and separation of the analyser crystals. (*a*) Using ∼5 keV X-rays, the gap between analyser crystals must be large enough (interval 8 mm, thickness 3 mm → gap 5 mm) and their length small enough to allow the passage of adjacent beams; at low energy, filtered beams are more easily partially cut by adjacent analyser crystals. (*b*) Using 9.5 keV X-rays, a crystal length of 26 mm allows filtering of 4.6 mm-wide beams. (*c*) Using ∼40 keV X-rays, a crystal length of 26 mm allows filtering of 1.6 mm-wide beams; the length of the analyser crystals should be longer (*e.g.* 80 mm) to allow filtering of a reasonable beam width (*e.g.* 4.6 mm).

So experimental powder diffraction beamlines using MAD arrays have been developed in SR facilities, because the flux of these sources is intense, and also because their energy can be chosen and their optical system optimized. To easily obtain impacts of the incident beam on the middle of several analysing crystals, a MAD filtering system has a filtering surface which corresponds to a logarithmic spiral (‘LogSpiral’) mathematical curve (Sakayanagi, 1982 ▸; Wittry *et al.*, 1993 ▸; Zhong *et al.*, 1999 ▸; Toraya, 2009 ▸). The characteristics of such a LogSpiral curve are that any analyser crystal located on it always sees its source/sample at the same Bragg angle Θ_A_, as shown for Si(111) analyser crystals in Fig. 1 ▸ and in Part 1 of this contribution (Prat & Hodeau, 2023 ▸; hereafter ‘Part 1’). So it would be best to build a rigid MAD system where, for a different set of experiments, the only parameters that can vary are the analyser angle Θ_A_ and the detector angle 2Θ_A_. As the Bragg angle Θ_A_ is imposed by the lattice distance *d*_*hkl*_ of the (*hkl*) crystal analyser and by the energy of the X-ray beam used for the experiment, the positioning of these analysers + detectors are determined by the choice of an optimal energy range. These parameters also determine the length of the analyser crystals and their minimum separation, as shown in Figs. 1 ▸(*a*)–1(*c*) for three energies with the same distance *D* between the sample and the MAD filter. With low-energy X-rays [Fig. 1 ▸(*a*)], the space between the analyser crystals must be large enough to allow the beams to pass. On the contrary, with high-energy X-rays [Fig. 1 ▸(*c*)], it is the length of the analyser crystals that must be large enough to allow the filtering of a beam of reasonable width. Indeed, with a distance *D* = 460 mm and 14 mm-long analyser crystals, the width of the beams (and therefore their intensity), filtered by such a MAD system with a pitch of 1°, goes from 4.6 mm at 5 keV to 2.6 mm at 9.5 keV and it is much thinner at 40 keV. If we increase the length of the crystals (*e.g.* to 26 mm), the maximum width of the filtered beams is 1.6 mm at 40 keV, which increases to 4.6 mm at 9.5 keV [Fig. 1 ▸(*b*)], but at lower energies the experiment is impossible because the beams are partially cut by the adjacent analyser crystals. Variations in beam widths/paths are more important when the MAD filtering system is designed to be operated at low energies. From these constraints, it is preferable to choose a high-energy range of the X-ray source to develop a multi-analyser diffraction system and to define its angular pitch between each analyser, its geometry, its size and finally its number of filtered channels.

As it is important not to limit ourselves to high-resolution measurements on SR sources, in this Part 2 we have investigated and tested a configuration that allows a small rigid MAD block to be used also for high-resolution powder diffraction analyses with laboratory X-rays sources. For this purpose, we have developed a configuration that seems to us to be the most optimal: use of MAD optics containing numerous Si(111) crystals working in reflection mode according to a geometry optimized for X-rays of energies close to that emitted by a laboratory Ag *K*α_1_ X-ray source. Indeed, such a high energy (22 keV) makes it possible to benefit from the advantages of the low diffraction angle Θ_A_ of the Si(111) planes. Our choice of Si(111) single crystals is guided by their good reflectivity which is associated with good angular resolution. The association of this MAD system with a Soller-collimator array is also fundamental to increase the signal-to-noise ratio of the measurements and thus to take advantage of the corresponding qualities of the 1D–2D photon-counting detectors. As reported in Part 1 ▸, such a small rigid MAD block can be realized by creating an array of 20–50 Si(111) crystals inserted in a rigid ‘comb-sandwich’ block, but also by a specific design/fabrication of a ‘single-crystal-comb’ block curved along a logarithmic spiral surface.

## 2. Specifications of the compact rigid multi-analyser

As the number of beams analysed is proportional to the crystal density of such a rigid system, it is important to have a relatively small angular pitch between them. The lower limit of this pitch is given by the manufacturing precision of the system: (i) a simple sandwich-comb of crystals, easily made by 3D printing, allows a pitch of 0.2° to be reached but not a pitch of 0.1°, (ii) a single-crystal-comb block having a rigid LogSpiral curvature, more difficult to build, enables a pitch of 0.1° to be achieved, as it offers higher precision and regularity of the orientations of each single-crystal plate of the block (Prat & Hodeau, 2023 ▸).

**Figure 2:**
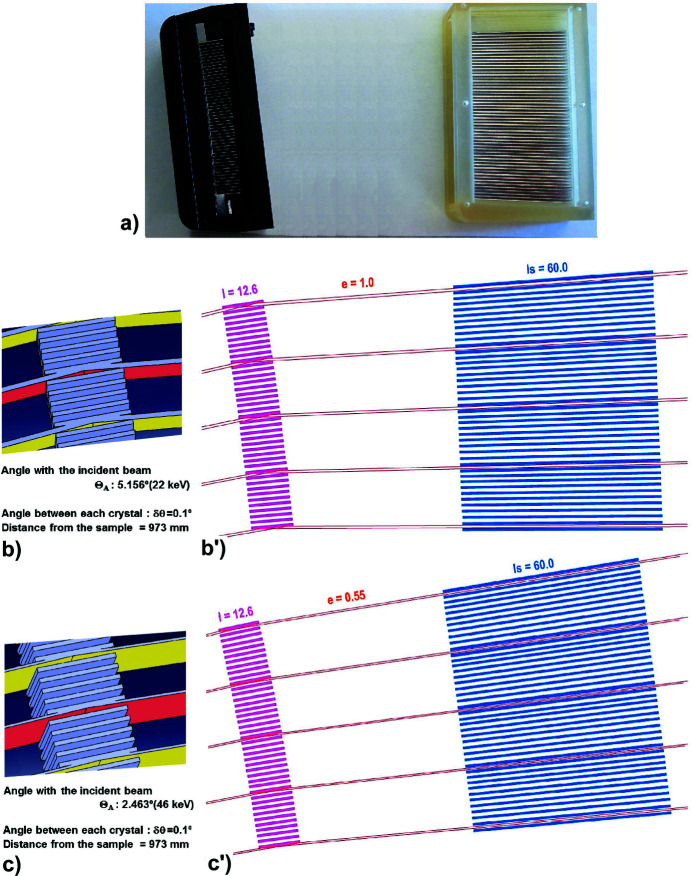
Representations of the components of the MAD system using a single-crystal-comb block and a Soller-collimator block which are optimized to filter X-ray beams in the 22–46 keV energy range: the energy of the X-rays determines the width of the impact of the filtered beams on the Si(111) crystals, the length of the Si(111) blades defines the width *e* of the filtered beams. Representation of the paths of the beams filtered by this MAD system having a pitch of 0.1°: the single-crystal-comb is at *D* = 973 mm from the sample and the period between each analyser crystal is 1.7 mm (crystal thickness 0.5 mm + space 1.20 mm), the Soller-collimator block is at *D*_s_ = 1033–1093 mm from the sample and the distance between the absorbing blades is 1.82 to 1.88 mm (see Part 1 ▸). (*a*) Photographs of the small single-crystal-comb and the Soller-collimator blocks; the comb is mounted on the goniometer Θ_A_ axis and the Soller-collimator block is mounted on the 2Θ_A_ arm of the goniometer. (*b*,  *b*′) Impact of the filtered beams on the Si(111) crystals with 22 keV X-rays, corresponding to Θ_A_ = 5.1556° and to beam width *e* = 1.0 mm; paths of these beams filtered by this MAD system have a pitch of 0.1°. (*c*, *c*′) Impact of the filtered beams on the Si(111) crystals with 46 keV X-rays, corresponding to Θ_A_ = 2.4632° and to beam width *e* = 0.55 mm; paths of these beams filtered by this MAD system have a pitch of 0.1°.

The results presented below relate to experiments carried out with such a rigidly curved single-crystal-comb block allowing a 0.1° pitch between 20–50 Si(111) crystals. This single-crystal MAD block is associated with a Soller-collimator block containing long collimators (60 mm) with the same angular pitch (0.1°) [Fig. 2 ▸(*a*)]. These two blocks were made using a multi-jet printing apparatus – ProJet MJP 3600: (i) the tungsten blades of the Soller block are placed in the slots of a 3D polymer holder, and (ii) the MAD block defines the LogSpiral curvature of its single-crystal-comb which is obtained by setting it on the corresponding curved surfaces of a second 3D polymer holder. Thanks to this process and to an *ad hoc* adjustment, a very good (±0.01°) accuracy is obtained for the alignment of the Si(111) crystals and allows a comb of perfect single crystals having a pitch of 0.1° to be produced, and this at a non-negligible but reasonable cost (about EUR 10000 in silicon).

The design of such a fixed MAD system requires that each beam cannot be cut by one of the adjacent analyser crystals. Therefore, to meet this requirement, we have designed a MAD system that can be used over a wide high-energy range (22 keV to 46 keV). Its distance to the sample under study was chosen to be large (*D* = 973 mm) to allow a small angular pitch (0.1°) between Si(111) crystals, corresponding to a distance between them of 1.7 mm. If the operating range allowed by the MAD system had been between 5 and 20 keV, the large variation in the impact of the beam on the analysers would have imposed a large interval distance between each analyser and a tenfold increase in angular pitch. For the wide high-energy range (22–46 keV) chosen for our system, the Θ_A_ angle of the beam on the Si(111) crystals is relatively small and grazing (5.156°–2.463°), which imposes a relatively long analyser crystal length (12.6 mm) [Figs. 2 ▸(*b*) and 2(*c*)].

The alignment and angle between the analyser crystals and the downstream Soller collimators varies according to the diffraction angle Θ_A_ of the analyser. This requires the Soller collimator to be rotated so that its position can be adjusted/optimized when there is a change in energy. The size of the fan aperture of the beams entering the Soller collimator also varies with the distance of this collimator from the sample. The MAD block is mounted on the Θ_A_ axis of the diffractometer arm so as to have its Si(111) diffraction planes parallel to this axis; this MAD system must be rigid because its alignment must be maintained regardless of the Θ_A_ angle. As it is essential that all analyser crystals and Sollers blades are correctly oriented, the Soller-collimator block is mounted with a goniometer head just before the 2D detector on the 2Θ_A_ axis of the diffractometer, with its central channel aligned with the central filtered beam path of the MAD block and with its input face perpendicular to this central beam path [Figs. 2 ▸(*b*′) and 2(*c*′)]. Small vertical/horizontal translations/rotations allow this Soller block to be perfectly adjusted to associate the path of each filtered beam to a collimator. As reported in Part 1 ▸, the images of the beam directly seen on the 2D detector greatly facilitate the optimization of the positions/orientations of the MAD block and the Soller-collimator block before stiffening their alignment [Fig. 7(*d*) of Part 1 ▸]. This adjustment should only be made when the energy of the X-ray source changes. For the two extreme energies 22 keV and 46 keV of the chosen usable range, these two blocks were designed to be identical and to be placed at the same distance from the sample; only their position/rotation corresponding to Θ_A_ and 2Θ_A_ angles varies (Θ_A_ = 5.1556° at 22 keV and Θ_A_ = 2.4632° at 46 keV) [Figs. 2 ▸(*b*′) and 2(*c*′)]. Thanks to this simple adjustment process, a very good alignment accuracy can be achieved, allowing the use of this comb of perfect single crystals with a pitch of 0.1°.

**Figure 3:**
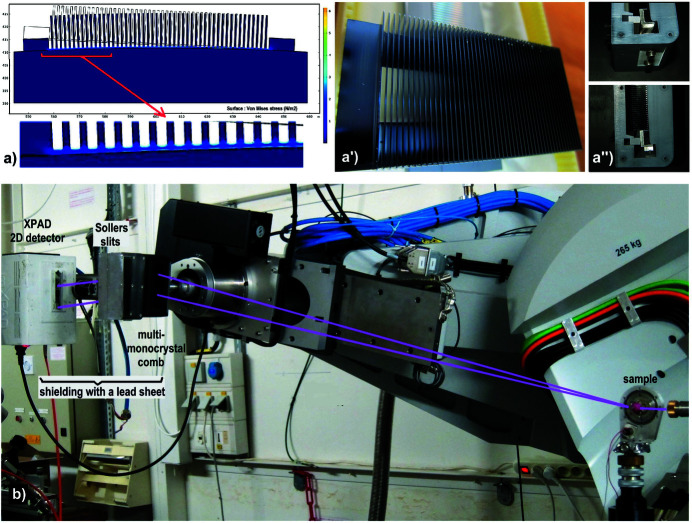
Photographs of the components of the MAD system using the single-crystal-comb and Soller-collimator blocks which are optimized to filter X-ray beams in the 22–46 keV energy range and photograph of their installation on the goniometer of the D2AM beamline. (*a*) Distribution of stresses in the single-crystal-comb during its bending (no stresses → many stresses corresponds to dark blue → light blue → green → yellow → orange); stresses are always less than 3 N m^−2^ (only blue colours) – the hole located under the Si(111) blades of this block has greatly reduced these stresses. (*a*′) Photograph of this single-crystal-comb cut in a single-crystal block of silicon and generating 50 single-crystal Si(111) blades – see also its hole located under all Si(111) blades. (*a*′′) This single-crystal-comb is adjusted with a fixing system on a rigid 3D printed support which imposes its LogSpiral curvature generating the orientations of the Si(111) crystal blades with a 0.1° pitch; enlargement of this fixing system. (*b*) Installation of this small MAD system on the Θ_A_ axis of the D2AM beamline goniometer, with the Soller-collimator block and the 2D photon-counting detector on the corresponding 2Θ_A_ arm; once the energy has been chosen, it is important to place a shielding lead sheet between the single-crystal-comb, the Soller-collimator block and the 2D detector (see the note on the left side of the photograph) in order to protect the filtered beam paths from environmental background noise; this photograph was taken before the installation of this lead shielding sheet.

This single-crystal-comb was cut directly from a single-crystal block of silicon, its size (110 mm) allowing the design of 50 strictly parallel Si(111) single-crystal blades spaced 1.7 mm apart [Figs. 3 ▸(*a*) and 3(*a*′)]. This comb is then bent to the desired logarithmic spiral curvature. As this bending could induce crystal stresses and some additional anticlastic curvature (Sparks *et al.*, 1982 ▸; Batterman & Berman, 1983 ▸; Ferrer *et al.*, 1992 ▸; Hazemann *et al.*, 1995 ▸), the single-crystal-comb design contains a thin bond and a large hole under each crystal [Fig. 3 ▸(*a*′)] which allows the stresses on the crystal blades to be relaxed as the comb is bent by its supporting block [Fig. 3 ▸(*a*)]. The supporting MAD block contains a small mechanical fixing/stiffness system that allows the curvature of the single-crystal-comb to be optimally adjusted to the LogSpiral curvature of the support [Fig. 3 ▸(*a*′′)]. The efficiency of this device was tested by measurements made on a LaB_6_ reference sample using the goniometer of the ESRF D2AM beamline [Fig. 3 ▸(*b*)] (Ferrer *et al.*, 1998 ▸; Basolo *et al.*, 2007 ▸; Chahine *et al.*, 2021 ▸).

## 3. Signal-to-noise ratio, intensity and resolution gains for powder diffraction experiments using a single-crystal-comb

The measurements and results presented below were collected with a quasi-parallel beam of 0.4 mrad using this rigid curved single-crystal-comb block associated with the Soller-collimator block, both having a 0.1° pitch. The experiment energy was 22 keV in order to easily extrapolate its potential gains to an experiment that could be performed (i) on a conventional source with an Ag *K*α_1_ X-ray tube in order to perform measurements in a laboratory or directly at an investigation site, or (ii) on a compact inverse Compton scattering X-ray source based on the power of lasers and the sharpness of optical cavities, which can provide an intense and thin beam accessible to laboratories at a cost one or two orders of magnitude lower than that of SR sources (Variola *et al.*, 2009 ▸; Jacquet, 2016 ▸; Eggl *et al.*, 2016 ▸; Hornberger *et al.*, 2019 ▸; Dupraz *et al.*, 2020 ▸).

The single-crystal-comb is mounted on the Θ_A_ axis of the D2AM beamline diffractometer at a distance *D* = 973 mm from the LaB_6_ sample and the Soller-collimator block is mounted with the 2D detector on its 2Θ_A_ arm. Fig. 3 ▸(*b*) highlights the small size of this compact MAD system. The 2D detector is an XPAD S70 hybrid pixel photon-counting 2D detector which consists of a succession of seven chips each containing 80 × 120 pixels; the pixel size is 125 µm × 125 µm and the used distance *D* > 1200 mm corresponds to an angular precision of 0.006° per pixel (Basolo *et al.*, 2007 ▸). In order to take full advantage of the background reduction achieved by the diffraction filtering, an additional shielding with a lead foil was placed between the single-crystal-comb, the Soller-collimator block and the 2D detector [Fig. 3 ▸(*b*)].

**Figure 4:**
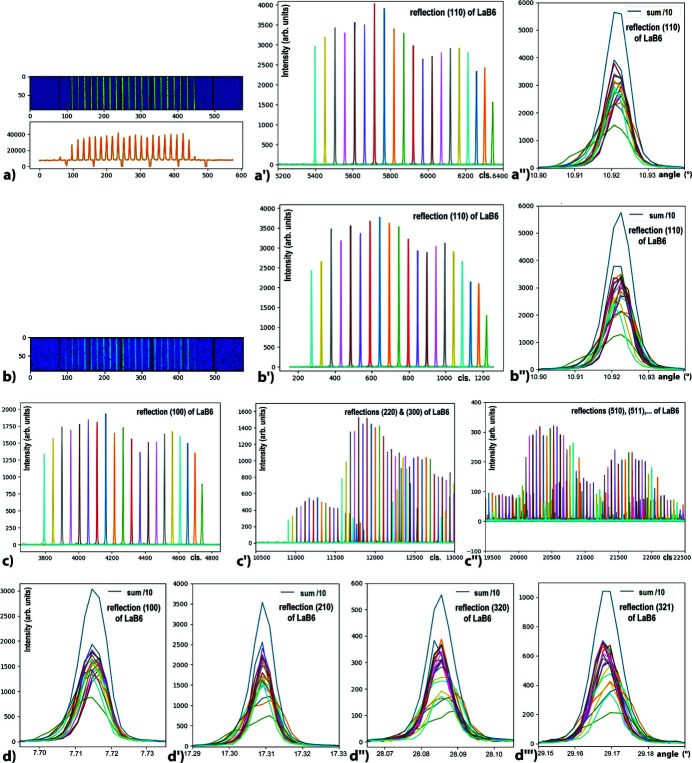
Intensities of diffraction reflections of a LaB_6_ reference powder, collected on the D2AM beamline goniometer with a 0.4 mrad quasi-parallel beam of size 80 µm × 150 µm and an energy of 22 keV, filtered by the two single-crystal-comb and Soller-collimator blocks, with an angular pitch of 0.1° between the Si(111) crystals. (*a*) Signals from the (110) reflection of LaB_6_, received by the XPAD S70 detector, which visualizes the 20 beams filtered by the single-crystal-comb. (*a*′) Intensities of the 20 signals filtered by each Si(111) analyser crystal for this (110) reflection (with a different colour for each analyser crystal). (*a*′′) Superposition of these 20 filtered intensities and their sum (divided by 10). (*b*, *b*′, *b*′′) Signals received by the 2D detector and profiles of these 20 beams filtered by each Si(111) analyser crystal for the same (110) reflection which was measured several days later, after data collections made with the same system on several other samples; we note a good constancy of the intensities of each of the beams filtered by each analyser crystal. (*c*, *c*′, *c*′′) Intensities of the diffracted lines filtered by each Si(111) analyser crystal at different Bragg angles corresponding to the LaB_6_ reflections: (*c*) (100), (*c*′) (220) and (300) and to (*c*′′) weak high angles reflections. (*d*, *d*′, *d*′′, *d*′′′) Superpositions of the 20 diffracted intensities filtered by each Si(111) analyser crystal of the single-crystal-comb, and their sum (divided by 10), for the LaB_6_ reflections (100), (210), (320) and (321).

The 2D images of the beams received by the 2D detector visualize 20 or so beams filtered by the single-crystal-comb [Figs. 4 ▸(*a*), 4(*a*′) and 4(*a*′′)]; these images also allow us to more easily optimize the alignment of the position/orientation of the Soller-collimator block before its stiffening. As shown in this figure, we have a very good parallelism and regularity of the angular alignment of the Si(111) blades of the single-crystal-comb, which makes it possible to precisely define very thin detection windows on the 2D detector and therefore to have a very low background noise and a very good separation of the 20 beams filtered by each analyser crystal with an angular pitch of 0.1°.

Comparison of Figs. 4 ▸(*a*), 4(*a*′) and 4(*a*′′) and Figs. 4 ▸(*b*), 4(*b*′) and 4(*b*′′) illustrates the almost identical distribution of intensities and beam positions obtained between a first measurement of the (110) reflection of LaB_6_ and a second measurement made a few days later on the same reflection after assembly/disassembly of the LaB_6_ sample. This second measurement was collected after several sample changes and after data collections made on several cultural heritage samples at the same energy; these data illustrate the alignment stability of this MAD filter that uses a single-crystal-comb. This stability is important because it allows the measurement of about 20 diffractograms in parallel with a constant contribution from each analyser crystal: even if they show some deviations in intensity, these constant measurement ratios allow all spectra/samples to be measured under the same conditions and thus to be corrected by an *ad hoc* data processing, as we had observed in 1995 when the first multiple analyser system was set up at ESRF (Hodeau *et al.*, 1996 ▸).

Figs. 4 ▸(*a*)–4(*d*) show profiles of the 20 single-crystal-comb contributions for several LaB_6_ reflections at low and high angles. In the outer parts of the comb, some lower intensities and asymmetric broadenings are observed and correspond to a partial measurement of the diffraction cone. These constant distortions can be related to a possible tilt of some of the Si(111) crystals or, more probably, to an imperfect residual adjustment of (i) the single-crystal-comb on the MAD block, (ii) the distances between the MAD-comb/Soller blocks and the sample, or (iii) their relative orientations on the Θ_A_/2Θ_A_ circles. As these residual beam intensity variations, observed in the thin detection windows defined on the 2D detector, are constant for the different experiments at a given energy, such effects can also be taken into account and corrected by data processing of the kind recently carried out on the ESRF ID22 beamline for analysis of diagrams collected with a 2° pitch MAD system associated with a 2D photon-counting detector (Dejoie *et al.*, 2018 ▸; Fitch & Dejoie, 2021 ▸).

**Figure 5:**
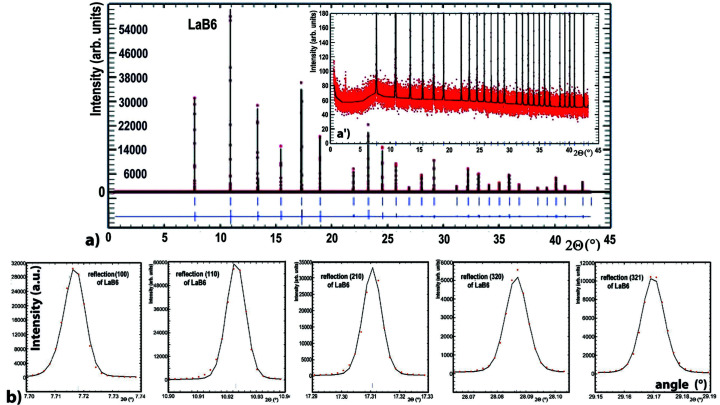
Diffraction pattern, and its Rietveld refinement, of an LaB_6_ reference powder using data filtered by the single-crystal-comb + Soller-collimator + photon-counting detector setup. (*a*, *a*′) Intensity of the complete diffractogram using the sum of the beams filtered by the 20 Si(111) single-crystal blades, and magnification of this diagram which underlines the low residual background of this measurement. (*b*) Visualization of the good agreement between measured and calculated profiles of the (100), (110), (210), (320) and (321) reflections of LaB_6_; their profiles are well described by a convolution of Gaussian and Lorentzian curves with FWHM = 0.008°.

Using this single-crystal-comb + Soller-collimator + photon-counting 2D detector setup we have collected the complete LaB_6_ diffractogram in ∼9 h with a 0.002°/1.5 sec step size/time (a continuous scanning mode can also be used). Figs. 5 ▸(*a*) and 5 ▸(*a*′) show the complete data and enlarge the residual noise of this pattern of a LaB_6_ reference powder collected on the D2AM beamline with a 0.4 mrad quasi-parallel beam, using the filtering of 20 analyser crystals of the single-crystal-comb. The Rietveld refinement of the intensity profiles, performed with the *FullProf* program (Rietveld, 1969 ▸; Rodríguez-Carvajal, 1993 ▸; Roisnel & Rodríquez-Carvajal, 2001 ▸), can be carried out with the Pearson VII pseudo-Voigt line profiles as variables (Hall *et al.*, 1977 ▸; Thompson *et al.*, 1987 ▸); it gives *R*_Bragg_ and *R*_f_ factors of 1.498 and 1.164, respectively. The peak widths of LaB_6_ reflections are 0.008°, their shape is a convolution of Gaussian and Lorentzian curves (η = 0.19) [Fig. 5 ▸(*b*)]. The intensity of the strongest LaB_6_ reflection is 60000 counts s^−1^ while the residual background is 60 counts s^−1^ [*i.e.* 3.0 counts s^−1^ per Si(111) channel!], which illustrates the very good signal-to-noise ratio provided by this small compact system.

Using the same alignment and adjustment of the single-crystal-comb + Soller-collimator blocks of this MAD system, we collected powder diffractograms of several complex heterogeneous samples from cultural heritage studies of the ‘Patrimalp’ project (Patrimalp, https://patrimalp.univ-grenoble-alpes.fr; Martinetto *et al.*, 2021 ▸; Bordet *et al.*, 2021 ▸; Autran, 2021 ▸; Autran *et al.*, 2021 ▸). The analysis of these cultural heritage materials is a real challenge for crystallography and, in the following experiment, illustrates the power of our MAD filter system, as they are very heterogeneous materials containing mixtures of mineral and/or organic compounds, corresponding to several crystallized and/or amorphous phases.

**Figure 6:**
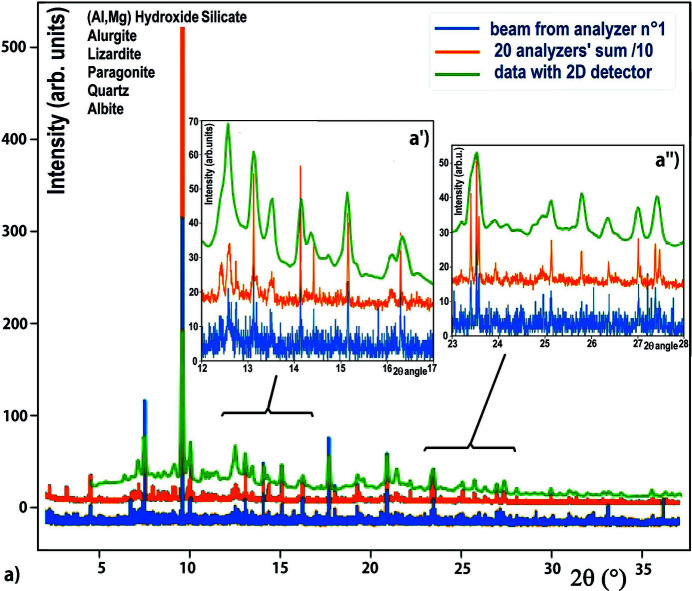
Diffraction pattern of a complex multiphase sample from Rocher du Château, a site in the Alps with schematic rock paintings attributed to the Neolithic period, using data collected with an 80 µm × 150 µm quasi-parallel beam on the D2AM beamline and filtered with the single-crystal-comb + Soller-collimator + 2D photon-counting detector setup, and compared with data collected with unfiltered 2D detector (green curve). (*a*) Full diffractogram of this cultural heritage sample with its measurements with an unfiltered 2D detector (green curve), with a single-crystal analyser (blue curve) and using the 20 beams filtered by the single-crystal-comb (orange curve). (*a*′, *a*′′) Enlarged diffractograms evidence the different phases of this cultural heritage sample which contains about ten phases, almost all silicates.

For these experiments, we first used a sample from Rocher du Château, a site in the Alps in Savoy-France with schematic rock paintings attributed to the Neolithic period and located on a high serpentinite cliff. This site shows rock paintings composed of a group of eight red deer and of some schematic figures (grids, dots,…); a first report of all the paintings was made in 1975 (Nelh, 1976 ▸) and they were studied in detail by Defrasne *et al.* (2019 ▸). In this last study, the surface information of the archaeological drawings was evaluated in detail by non-invasive *in situ* methods (digital microscopy and Raman spectroscopy), and some micro-samples were analysed by scanning electron microscopy energy-dispersive X-ray spectroscopy (SEM-EDX) and X-ray diffraction (XRD) methods. We used one of these micro-sampling units to test the sensitivity of our MAD filter by performing an XRD experiment on it, with a quasi-parallel beam of 0.4 mrad at an energy of 22 keV. The corresponding powder diffractogram (Fig. 6 ▸), collected using Debye–Scherrer geometry in ∼4 h with a step size/time of 0.004°/1.5 sec, illustrates the richness/complexity of such samples that contain the signatures of various materials. The green curve in Fig. 6 ▸ corresponds to a direct data collection using a conventional 2D detector, the orange curve corresponds to the data collection using the MAD filtering using the 20 analyser crystals of the single-crystal-comb and the Soller-collimator block, and the blue curve corresponds to the signal received by a single analyser. It is obvious that the diffraction filtering has a very weak intensity response compared with that of the 2D detector, but with a single 2D detector the background signal is high and the line widths are so wide that a peak may contain one, two, three reflections…, so we cannot differentiate the diffracted lines and the crystallinity of these different phases [Figs. 6 ▸(*a*′) and 6(*a*′′)]. This powder pattern reveals about ten phases, the majority of which are phyllosilicates, already observed by Defrasne *et al.* (2019 ▸), which could be derived from the serpentine bedrock substrate, the most important being: chamosite (Mg_5_Fe_5_)Al_2.7_(Si_5.7_Al_2.3_)O_20_(OH)_16_, paragonite NaAl_2_(AlSi_3_)O_10_(OH)_2_, alurgite (K_1–*x*_Na_*x*_)[Al_1–*y*_(Mg,Fe,Mn)_*y*_]_2_(AlSi_3_)O_10_(OH)_2_, lizardite Mg_3_(Si_2_O_5_)(OH)_4_, albite (NaAlSi_3_O_8_), quartz (SiO_2_)…. In contrast to the diagram resulting from the sum of the 20 beams filtered by the single-crystal-comb (orange curves), which allow the collection of weak refections, the diagram measured and filtered by only a single analyser crystal (blue curves) is really too weak to distinguish the weak signals that contain rich information on the minor phases.

**Figure 7:**
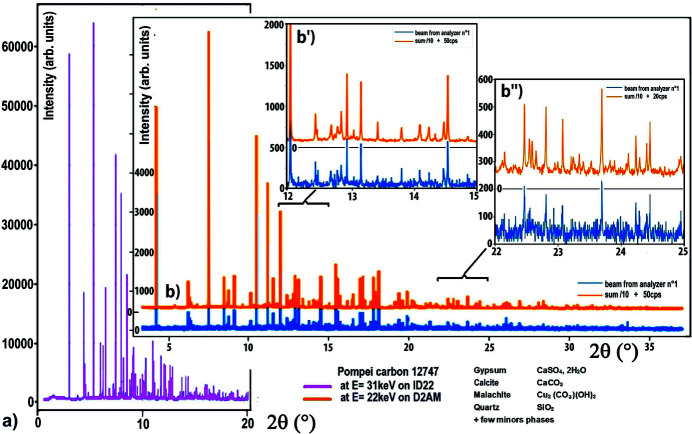
Comparison of powder diffractograms of archaeological samples found in the same bronze vessel from Pompeii, which were collected on the high-resolution powder ID22 beamline and on the D2AM beamline using the single-crystal-comb + Soller-collimator blocks + 2D photon-counting detector setup. (*a*) Diffractogram collected on the high-resolution powder beamline ID22 (pink curve). (*b*) Diffractogram collected on the beamline D2AM using the single-crystal-comb system; the blue curve corresponds to data from one single central channel of this single-crystal-comb and the orange curve uses the sum of its 20 channels. (*b*′, *b*′′) Enlarged areas of this pattern highlight the mixture of a dozen phases including gypsum, calcite, malachite and quartz.

The second cultural heritage sample we used to test the single-crystal-comb filtering system is a black powder from vessels found in houses in Pompeii. These black powders were chosen for their mention in ancient texts (Littre, 1855 ▸; Discorides, 2000 ▸) and for their possible artistic uses in antiquity; they were therefore studied to specify such uses (Tomasini *et al.*, 2012 ▸; Cersoy *et al.*, 2016 ▸). These samples contain pigments that can correspond to various uses: inks, cosmetics, paints. Fig. 7 ▸(*b*) shows the full diffraction pattern of a powder contained in a bronze container, collected with a single-crystal analyser and then using the 20 signals from the single-crystal-comb with a step size/time of 0.004°/1.5 sec. The two enlargements [Figs. 7 ▸(*b*′) and 7(*b*′′)] of this figure highlight the importance of the multi-filtering (×20) of the single-crystal-comb to generate gains in intensity and resolution that allow the visualization/quantification of different minor phase reflections. These powder diagrams show no diffuse scattering and there is a very good separation of the crystalline phases which allows their crystallinity to be estimated. Previous photographs and microanalysis of an identical sample coming from the same container showed its heterogeneity and revealed the presence of a mixture of coloured grains (dark grey, white, black and green) and a variety of compositions, notably in Ca, Cu and Si (Welcomme, 2007 ▸; Cersoy *et al.*, 2016 ▸). Figs. 7 ▸(*a*) and 7 ▸(*b*) compare two powder diffractograms collected using Debye–Scherrer geometry on two samples coming from this same vessel (*b*) with the MAD single-crystal-comb on the D2AM beamline, and (*a*) with the ID22 high-resolution powder diffraction beamline at ESRF (Autran, 2021 ▸; Autran *et al.*, 2021 ▸). They show a very good agreement, giving the proportions and crystallinities of a mixture of a dozen phases including gypsum (CaSO_4_.2H_2_O), calcite (CaCO_3_), malachite [Cu_2_CO_3_(OH)_2_] and quartz (SiO_2_). The malachite observed could be the result of degradation of the bronze vessel, the presence of gypsum and calcite having already been mentioned for the preparation of such inks and used in particular as a mineral filler to accelerate the drying process (Welcomme, 2007 ▸; Gamberini *et al.*, 2008 ▸; Canevali *et al.*, 2011 ▸). Comparison of the measurements made (*b*) with the MAD filtering setup on the D2AM beamline and (*a*) with the ID22 reference beamline for high-resolution powder diffraction highlights the excellent signal-to-noise ratio, the good resolution and the intensities obtained by using the filtering with the proposed combination of small rigid single-crystal-comb block + Soller-collimator block + 2D photon-counting detector. It underlines the interest of this system to carry out clean measurements of very heterogeneous ordered and/or disordered materials. Although the instrumental resolution of the MAD single-crystal-comb (FWHM ≃ 0.008°) is much larger than that of the ESRF ID22 powder diffraction reference beamline (FWHM ≃ 0.0023°) (Dejoie *et al.*, 2018 ▸; Fitch & Dejoie, 2021 ▸), the width of the diffraction peaks of each sample is related to the crystallinity of its phases, the fluorescence is suppressed and the experimental noise is very low; therefore, its use allows the detection and quantification of minor phases of such complex heterogeneous samples.

## 4. Conclusion

We have developed a multiple analyser system using diffraction designed to be combined with 1D or 2D detectors for accurate structural analyses. This MAD filter has the shape of a single-crystal-comb block curved on a support surface following a logarithmic spiral curve. This system consists of a rigid single-crystal analyser array which diffracts in reflection mode and contains 20–50 single Si(111) crystal blades accurately aligned (±0.01°), each filtering the sample signal with a 0.1° pitch when it is mounted on a long arm allowing a large distance *D* between the sample and the MAD multi-analyser + Soller + detector setup (of the order of 800–1000 mm). The most difficult part of the realization of this MAD filter is the cutting and polishing of the single-crystal-comb itself, which has a non-negligible cost. To simplify the manufacture of the MAD filter and reduce its cost, it is possible to replace the single-crystal-comb of this MAD system by a sandwich-comb (Part 1 ▸), but the lower precision in the orientations of the successive Si(111) crystals (±0.05°) will require the design of a system with only 10–25 single-crystal blades, filtering with a pitch of 0.2° for a device with the same large distance *D* between the sample and the MAD filter system.

The geometry of this rigid MAD filter was optimized to be used over a wide range of synchrotron X-ray high energies with an accurate analyser crystal alignment and was tested by data collection of complete diffractograms of a LaB_6_ reference powder and of a few complex cultural heritage samples. For these data collections, our rigid MAD filter associates a single-crystal-comb, a Soller-collimator and a 2D photon-counting detector, which provide excellent signal-to-noise ratios, reasonable intensity gains and narrow reflection profiles. These excellent results, obtained on the D2AM beamline at an energy of 22 keV using the ESRF source, could partly be extrapolated to laboratory experiments with an Ag *K*α_1_ tube X-ray source. The reflection profiles will probably be wider due to less efficient pre-sample parallel optics of the source/beam, and the intensities will be much lower due to the intensity of tube-type sources. In this case, the possible use of a comb composed of crystals with a larger mosaic/stresses would increase the filtered intensity but would decrease the resolution. The use of a 1D photon-counting detector instead of a 2D detector is possible, if it is chosen to filter only a narrow band of beams in the direction perpendicular to the Θ diffraction plane. In all the cases the gains obtained in intensity and mainly in signal-to-noise ratios are important and are strongly determined by the post-sample optics and therefore by the qualities of the combination of single-crystal-comb + Soller-collimator + 2D photon-counting detector.

The small size of the single-crystal-comb and Soller-collimator blocks also suggests that such a MAD filtering system could also be used to increase the resolution of some experiments using laboratory sources. The complete MAD-filtering system is small (less than 150 mm × 250 mm × 150 mm, with the addition of the 2D detector) and lightweight, allowing it to be mounted on laboratory equipment having a long 2Θ arm. Indeed, the main constraint to obtain a respectable intensity gain is that this MAD system must be rigid and able to insert 20–50 single-crystal blades, each filtering the sample signal with a 0.1° pitch, which imposes a large distance *D* between the sample and the multi-analyser + detector system (of the order of 800–1000 mm). If the geometry of the goniometer only allows a sample-to-detector distance of 400–500 mm, the MAD system will only be able to hold 10 to 25 single-crystal blades.

If the energy of the X-ray source is unique, as in the case for a laboratory device using only an Ag *K*α_1_ X-ray source, it is possible to reduce the variability of the settings by making a rigid system consisting of a single compact-rigid block that incorporates both the MAD comb and the Soller-collimator array. The final alignment of such a system would first need to be optimized and then rigidly fixed to the instrument during its assembly, prior to its experimental uses in the laboratory. In this case it might also be possible to use mosaic crystals instead of perfect Si(111) crystals to match the resolution offered by the other components of a laboratory source and to use a sandwich-comb block allowing filtering with a 0.2° pitch.

The benefits of such an improvement in the resolution of powder diffraction instruments/experiments and in particular in their signal-to-noise ratios are also essential for studies of mixed samples containing both crystalline and amorphous components. Although, after diffraction filtering, the measured intensities can be relatively low, the very good signal-to-noise ratio given by the MAD single-crystal-comb allows minority phases to be extracted and also the diffusion of amorphous phases to be quantified when they are present. This gain is essential for studies of complex heterogeneous materials such as ‘real’ materials of industrial type, cultural heritage, *etc.*, as these materials generally present difficult problems in identifying and quantifying their many minor phases.
